# Dual-labeled anti-GD2 targeted probe for intraoperative molecular imaging of neuroblastoma

**DOI:** 10.1186/s12967-024-05728-0

**Published:** 2024-10-15

**Authors:** Lauren Taylor Rosenblum, ReidAnn E. Sever, Ryan Gilbert, David Guerrero, Sarah R. Vincze, Dominic M. Menendez, Peggy A. Birikorang, Mikayla R. Rodgers, Ambika Parmar Jaswal, Alexander C. Vanover, Joseph D. Latoche, Angel G. Cortez, Kathryn E. Day, Lesley M. Foley, Chaim T. Sneiderman, Itay Raphael, T. Kevin Hitchens, Jessie R. Nedrow, Gary Kohanbash, W. Barry Edwards, Marcus M. Malek

**Affiliations:** 1 Department of General Surgery, University of Pittsburgh, 200 Lothrop Street, Pittsburgh, PA 15213 USA; 2 Department of Neurological Surgery, Children’s Hospital of Pittsburgh of UPMC, 7131 Rangos Research Building, 530 45th Street, Pittsburgh, PA 15201 USA; 3 University of Pittsburgh School of Medicine, 3550 Terrace Street, Pittsburgh, PA 15213 USA; 4 Department of Biochemistry, University of Missouri, 117 Schweitzer Hall, Columbia, MO 65211 USA; 5 In Vivo Imaging Facility Core, Hillman Cancer Center, University of Pittsburgh Medical Center, 5115 Centre Avenue, Pittsburgh, PA 15232 USA; 6 Department of Immunology, University of Pittsburgh Medical Center, 200 Lothrop Street, Pittsburgh, PA 15213 USA; 7 Department of Pediatric General Surgery, University of Pittsburgh Medical Center, One Children’s Hospital Drive, 4401 Penn Ave., Faculty Pavilion 7th Floor, Pittsburgh, PA 15224 USA

**Keywords:** Intraoperative molecular imaging, Neuroblastoma, GD2, Radio-guided surgery, Fluorescence guided surgery, Monoclonal antibody

## Abstract

**Background:**

Surgical resection is integral for the treatment of neuroblastoma, the most common extracranial solid malignancy in children. Safely locating and resecting primary tumor and remote deposits of disease remains a significant challenge, resulting in high rates of complications and incomplete surgery, worsening outcomes. Intraoperative molecular imaging (IMI) uses targeted radioactive or fluorescent tracers to identify and visualize tumors intraoperatively. GD2 was selected as an IMI target, as it is highly overexpressed in neuroblastoma and minimally expressed in normal tissue.

**Methods:**

GD2 expression in neuroblastoma cell lines was measured by flow cytometry. DTPA and IRDye^®^ 800CW were conjugated to anti-GD2 antibody to generate DTPA-αGD2-IR800. Binding affinity (Kd) of the antibody and the non-radiolabeled tracer were then measured by ELISA assay. Human neuroblastoma SK-N-BE(2) cells were surgically injected into the left adrenal gland of 3.5-5-week-old nude mice and the orthotopic xenograft tumors grew for 5 weeks. ^111^In-αGD2-IR800 or isotype control tracer was administered via tail vein injection. After 4 and 6 days, mice were euthanized and gamma and fluorescence biodistributions were measured using a gamma counter and ImageJ analysis of acquired SPY-PHI fluorescence images of resected organs (including tumor, contralateral adrenal, kidneys, liver, muscle, blood, and others). Organ uptake was compared by one-way ANOVA (with a separate analysis for each tracer/day combination), and if significant, Sidak’s multiple comparison test was used to compare the uptake of each organ to the tumor. Handheld tools were also used to detect and visualize tumor in situ, and to assess for residual disease following non-guided resection.

**Results:**

^111^In-αGD2-IR800 was successfully synthesized with 0.75-2.0 DTPA and 2–3 IRDye^®^ 800CW per antibody and retained adequate antigen-binding (K_d_ = 2.39 nM for aGD2 vs. 21.31 nM for DTPA-aGD2-IR800). The anti-GD2 tracer demonstrated antigen-specific uptake in mice with human neuroblastoma xenografts (gamma biodistribution tumor-to-blood ratios of 3.87 and 3.88 on days 4 and 6 with anti-GD2 tracer), while isotype control tracer did not accumulate (0.414 and 0.514 on days 4 and 6). Probe accumulation in xenografts was detected and visualized using widely available operative tools (Neoprobe^®^ and SPY-PHI camera) and facilitated detection ofputative residual disease in the resection cavity following unguided resection.

**Conclusions:**

We have developed a dual-labeled anti-GD2 antibody-based tracer that incorporates In-111 and IRDye^®^ 800CW for radio- and fluorescence-guided surgery, respectively. The tracer adequately binds to GD2, specifically accumulates in GD2-expressing xenograft tumors, and enables tumor visualization with a hand-held NIR camera. These results encourage the development of ^111^In-αGD2-IR800 for future use in children with neuroblastoma, with the goal of improving patient safety, completeness of resection, and overall patient outcomes.

**Supplementary Information:**

The online version contains supplementary material available at 10.1186/s12967-024-05728-0.

## Background

Neuroblastoma (NB) is the most common extracranial solid malignancy in children and accounts for 15% of pediatric cancer-related deaths [1]. Over 30% of NB patients have high-risk disease, which is associated with a 5-year survival of only 40–50% [2]. These poor outcomes are observed despite intense, multimodal therapy that includes surgery, high-dose chemotherapy, radiotherapy, autologous stem cell transplantation, and immunotherapy [3–5]. Tumor encasement of critical neurovascular structures, unclear tumor margins, and remote lymph node disease present particularly significant challenges for surgeons attempting safe and complete macroscopic excision of NB. In fact, up to 50% of patients experience significant surgery-related complications such as unplanned nephrectomies, Horner syndrome, pleural effusions, significant blood loss, or even death [6, 7]. In addition, up to 30% undergo inadequate surgery (defined as either less than 90% resection or less than complete macroscopic resection), which is associated with increased cumulative incidence of local progression and decreased event-free survival [6–8]. The challenge of safely separating cancer from critical structures that are encased in tumor and related attempts to avoid complications could also contribute to incomplete resection. Innovations to improve disease localization and clarify tumor margins intraoperatively should lead to safer operations with more complete resections, which in turn would lead to improved patient outcomes.

Approaches for aiding surgeons in the resection of cancer, such as radio-guided surgery (RGS) and fluorescence-guided surgery (FGS), have been developed over several decades and provide intraoperative surgical guidance for a number of tumors, though they have had limited translation for NB surgery. RGS utilizes gamma emissions from radioisotopes to localize regions of interest and is commonly used for sentinel lymph node biopsy in melanoma and breast cancer [9, 10]. This technique relies on the passive accumulation of radiotracer, but receptor targeted gamma-emitting probes are now being developed, including ^111^In-labeled antibodies targeting prostate, colorectal, renal cell carcinoma, and ovarian cancers [11]. While gamma decay is detectable through several centimeters of tissue, there are no visual cues to distinguish the lesion from adjacent tissue, so it is less useful for detecting surgical margins.

In contrast, FGS provides real-time visualization of lesions through a few millimeters of tissue. Initial FGS techniques also relied on passive accumulation of tracers, such as using indocyanine green (ICG) to highlight biliary anatomy, though this leads to significant background fluorescence in cancer applications, most notably in areas of inflammation and in benign lesions. To address the shortcomings seen with the passive accumulation of tracers, receptor-targeted fluorescent tracers have recently been developed. For example, an FDA-approved targeted fluorescent agent, OTL38 (also known as pafolacianine or Cytalux), targets the folate receptor and was approved in 2021 and 2022 for intraoperative imaging of ovarian and lung cancers [12, 13]. Antibody-based targeted fluorescent probes, such as SGM-101, which targets carcinoembryonic antigen (CEA) on colorectal cancer, have also shown promising results in clinical trials [14–16]. Despite the utility of tumor visualization, near-infrared (NIR) fluorescent signal can only penetrate through 5–10 mm of intervening tissue [14, 15]. 

Dual-labeled IMI agents combine the advantages of RGS and FGS, enabling both depth of detection for identification of occult lesions (with radioactivity) and tumor margin visualization (with fluorescence). Accordingly, the development of targeted dual-labeled probes has gained enthusiasm, with several agents targeting adult tumors now in the early stages of development, including ^111^In-DTPA-labetuzumab-IRDye800 which targets CEA for colorectal cancer, ^111^In-DTPA-bivatuzumab-IRDye800 which targets CD44 for head and neck cancer, and ^111^In-DOTA-girentuximab-IRDye800 for renal cell carcinoma [17–19]. While many of these dual-labeled tracers are promising, none could be used for to aid in surgical resection of neuroblastoma, as none have been FDA-approved nor are any designed for pediatric indications.

GD2 is a well-established tumor antigen, present on 98% of NB but minimal expression in normal tissues, and it is currently targeted by the monoclonal antibody, Dinutuximab, for standard maintenance immunotherapy for high-risk NB [5, 20–22]. In-111 decays by gamma emission with a half-life of 2.81 days, which is well-matched to the 1-to-4-week serum half-life of IgG antibodies, and it has a low energy emission enabling more specific detection than seen with higher energy counterparts [23, 24]. NIR fluorophores have less absorption by tissue and less overlap with tissue autofluorescence than visible spectrum fluorophores, enabling better tumor-to-noise signal and greater depth of visualization [25, 26]. IRDye^®^ 800CW is a near-infrared (NIR) dye with high brightness that has shown promise when conjugated to antibodies for FGS. Tracers that incorporate it are now in clinical trials for pancreatic, breast, colorectal, and head and neck cancers (NCT02736578, NCT01972373, NCT01508572, NCT04511078). The specificity of the anti-GD2 antibody for NB, the low-energy gamma decay and half-life of In-111, and the optical properties of IRDye^®^ 800CW make them well suited for a dual-labeled IMI agent.

Here, we demonstrate the ability of an anti-GD2 monoclonal antibody labeled with a gamma emitting radionuclide and a NIR fluorophore, ^111^In-αGD2-IR800, to enable IMI in an established orthotopic xenograft mouse model of neuroblastoma through antigen-specific accumulation.

## Methods

### In vitro maintenance of cell lines

NMB6 (Shou-Jiang Gao Lab for Cancer Virology, University of Pittsburgh), SK-N-BE(2) (ATCC no. CRL-2271), SK-N-AS (ATCC no. CRL-2137), SK-N-SH (ATCC no. HTB-11), SH-SY5Y (ATCC no. CRL-2266) and CHP212 (CHP tumor bank) cells were cultured in Dulbecco’s Modified Eagle Medium (Cat# BW12614F; Lonza, Basel, Switzerland), supplemented with 10% heat-inactivated FBS (Cat# SH3091003; Cytiva Hyclone™, Logan, UT, USA), 100 mM sodium pyruvate (Cat# BW13-115E; Lonza, Basel, Switzerland), 1x Antibiotic-Antimycotic (Cat #15-240-062; Gibco, Grand Island, New York, USA), 55 µM beta-mercaptoethanol (Cat# 21-985-023; Gibco), 1x MEM NEAA (Cat# 11140-050; Gibco), and 50 mg/mL Normicin (Cat# ant-nr-2; InvivoGen, San Diego, CA) and maintained in a 37 °C humidified incubator with 5% CO_2_. SK-N-BE(2), SK-N-AS, SK-N-SH, and SH-SY5Y cell lines were authenticated through genotypic analysis performed by ATCC.

### Flow cytometry and quantification of GD2 expression

Cells were detached from T-75 flasks (Cat# 430641U; Corning™, Corning, NY, USA) with 0.05% Trypsin / 0.53 mM EDTA in HBSS (Cat# MT25052CI; Corning™). A standard curve of fluorescence was created using BD Quantibright™ beads with known levels of PE staining on the LSR Fortessa FACS machine (BD Biosciences, Franklin Lakes, New Jersey, USA), as described in the PE Fluorescence Quantitation Kit (Cat# 340495; BD Biosciences). Each cell line was stained with 1 µg/1,000,000 cells PE anti-human Ganglioside GD2 (Cat# 357304; BioLegend^®^, San Diego, CA, USA) at 4 °C for 30 min. PE fluorescence was measured from at least 10,000 cells, then quantified with the linear regression of the generated standard curve. Data were analyzed using FlowJo Software, v10.8 (BD Biosciences).

### Tracer and isotype control synthesis and evaluation

To generate DTPA-aGD2-IR800 or DTPA-isotype-IR800, 1 mg of mouse IgG2a anti-GD2 (#BE0318; Bio X Cell, Lebanon, NH, USA) or mouse IgG2a isotype control (#BE0085; Bio X Cell) in PBS was added to 0.1 M sodium carbonate buffer, pH 9 (final pH 8.5-9). 25-fold molar excess of p-SCN-Bn-CHX-A”-DTPA (Macrocyclics, Plano, TX) was added (37 °C,1 h). Excess DTPA was removed by centrifugal ultrafiltration (Pierce Protein Concentrator, 10 kDa MWCO, PBS diluent, Thermo Fisher Scientific, Waltham, MA, USA). Four-fold molar excess of IRDye^®^ 800CW NHS ester (LI-COR, Lincoln, NE, USA) was added (final pH 8.5-9) and incubated (RT, 30 min, with shaking). After each conjugation, purification was performed with size exclusion HPLC (Agilent 1260, Bio SEC-3, PBS, 0.3 mL/min). DTPA substitution levels were calculated by comparing absorbance at 245 nm (DTPA) and 280 nm (protein), while IRDye^®^ 800CW substitution levels were determined comparing absorbance at 700 nm (IRDye^®^ 800CW) and 280 nm (protein with 3% from IRDye^®^ 800CW) by UV-Vis Spectrophotometry (BioTek Epoch with a Take3 plate; Agilent, Santa Clara, CA, USA). Probes were protected from light and stored at less than 3 mg/ml until use.

DTPA-αGD2-IR800 or DTPA-isotype-IR800 was radiolabeled with In-111 using the Nalla et al. method for radiolabeling DTPA-conjugated proteins with minimal modifications [27]. In-111 stock was diluted into sodium citrate (500 mM; pH 6) to 1.6 ml and after 5 min, probe was added and incubated (25 °C, 30 min). Reaction progress was monitored by instant thin-layer chromatography (iTLC) developed in 50 mM sodium citrate eluent. The buffer was exchanged with DPBS (Cat# 175512F12; Lonza, Basel, Switzerland) for tail vein injections. Purity was determined by SEC-HPLC (PBS, 3 μm, 300 A, 4.6 × 300 mm, 0.4 mL/min; BioSec3, Agilent), and purification repeated until > 95% pure.

To assess potential changes in GD2-specific binding affinity after conjugation of the dye and chelator, an ELISA was performed [28]. Briefly, ganglioside GD2 (Advanced ImmunoChemical Inc., Long Beach, CA, USA) was reconstituted (1 mg/ml) and immobilized on a microtiter plate (0.8 ug/well). After blocking with 1% BSA, a range of concentrations of anti-GD2 or DTPA-αGD2-IR800 were added (2.6–333 nM), followed by HRP-conjugated goat anti-human IgG Fc (Thermo Fisher Scientific, Waltham, MA, USA), then visualized. Nonspecific binding was determined with GD2 immobilization and secondary antibody only (HRP-conjugated goat anti-human IgG Fc), then total binding was corrected (by subtracting the non-specific binding) to yield specific binding, which was then fit by non-linear regression to yield the equilibrium dissociation constants (GraphPad Prism v9.4.1, Boston, MA, USA).

## In vivo studies

### Orthotopic xenograft mouse model

Animal studies were performed in accordance with the protocols approved by the Institutional Animal Care and Use Committee of the University of Pittsburgh (IACUC protocol number: 19126346). Four- to six-week-old nude mice (*Fox1*^*nu*^, strain #002019; The Jackson Laboratory, Bar Harbor, ME, USA) were maintained in a temperature-controlled animal facility at the UPMC Children’s Hospital of Pittsburgh or the UPMC Hillman Cancer Center with a 12-hour light/dark cycle in cohorts of five. Animals were kept in the facility for at least two days prior to performing any procedures to minimize stress-related symptoms.

Tumors were grafted by the established adrenal gland injection method [29]. Under isoflurane anesthesia, a transverse left flank incision was made though skin, abdominal wall, and peritoneum. The left kidney and adrenal gland were gently exteriorized and maneuvered by palpation and with a cotton tip applicator. SK-N-BE(2) cells were trypsinized, washed, counted, and resuspended in PBS. One million SK-N-BE(2) human-derived neuroblastoma cells in 20 µl of PBS were mixed with 20 µl of Matrigel basement membrane matrix (#354234; Corning™, Corning, NY, USA), then injected into the left adrenal gland with a 30-gauge needle on a 1 ml syringe and the needle was slowly removed, with the Matrigel holding the cells largely in place. The kidney and adrenal gland were returned to the abdomen, the body wall was closed with 3 − 0 or 4 − 0 Polysorb, and the skin was closed with surgical wound clips (which were removed after 10–14 days). Mice were placed in a clean cage on a warming pad provided with easily accessible food on the cage bottom, and monitored at least every 15 min until sternal recumbency was regained and they had fully recovered from anesthesia.

Tumor size was measured by MRI 5 weeks after tumor injection (7T/30-cm AVIII spectrometer, Bruker Biospin, Billerica, MA; 12 cm gradient set, 40 mm quadrature RF volume coil, and Paravision 6.0.1 software). A T1-weighted Intragate FLASH sequence was used (repetition time (TR)/echo time (TE) = 162/2.6 ms, field of view 40 × 40 mm, acquisition matrix 256 × 256, 13 slices, slice thickness 1 mm, and 1 average). Tumor size on MRI images was measured using DSI Studios software. Euthanasia was planned for any animals that reached USDA Class D sacrifice requirements, though none did prior to planned euthanasia. All mice grew tumors and were included in the study. Mice were excluded from analysis for clear and uncorrectable data errors (multiple organs with uptake and weight more than 2 standard deviations from others in the group).

### Handheld gamma detection, fluorescence detection, and biodistribution studies

Mice were used for further experiments 3.5-5 weeks after SK-N-BE(2) injection, when tumors were approximately 0.5–1 cm large (*n* = 33 in total, including 26 for anti-GD2 tracer groups and 7 for isotype control groups). For each experiment, mice were randomly assigned to each group.

On the day of radiolabeling, ^111^In-αGD2-IR800 (*n* = 7 for day 4 and *n* = 7 for day 6) or ^111^In-isotype-IR800 (*n* = 4 for day 4 and *n* = 3 for day 6) was injected intravenously by tail vein (0.5–4.8 MBq/50 µg in 100µL DPBS). Three and four days after tracer injection, gamma counts from the day 4 tracer group were measured percutaneously from the left flank (tumor), right flank, left and right hind limbs, tail, and chest using a Neoprobe^®^ gamma decay detector (Mammotome, Cincinnati, OH, USA). Four or six days after tracer or isotype control injection, mice were euthanized for gamma biodistribution analysis. NIR imaging was performed with the SPY-PHI NIR camera (Stryker, Leesburg, VA, USA) post-laparotomy, with representative images recorded.

Personnel performing injections and biodistribution studies were not blinded as to whether probe or isotype control was administered. The indicated organs were harvested and weighed (liver piece, spleen, tumor, blood, muscle piece, femur bone piece, right adrenal, left kidney, right kidney, heart, lung, section of small intestine, and brain). Tissue-associated radioactivity was measured by gamma counter (PerkinElmer Wizard 2, model 2480; PerkinElmer, Waltham, MA, USA) and quantified as percent injected dose (corrected for In-111 decay from the dose measured at the time of injection) per gram of tissue (%ID/g). One mouse was removed from the study (in the day 6 ^111^In-αGD2-IR800 gamma biodistribution group) because of an uncorrectable data error (leading to *n* = 6 for that group).

For the optical biodistribution, additional tumors were grown (*n* = 10) and after 4.5 weeks,^111^In-αGD2-IR800 was injected intravenously (6.3 MBq/74.6 µg in 100µL DPBS). Mice were euthanized 4 or 6 days later (*n* = 5 each). Organs were harvested and placed on a minimally fluorescent black PLA printed plate, and images were acquired with the SPY-PHI camera, and saved using a NIX HDMI capture card (model USBC-CAP60, Plugable Technologies, Redmond, WA, USA) and OBS video capture software (Open Broadcaster Software, open source). To define regions of interest (ROIs) for each organ, the brightness and contrast were uniformly enhanced on each image to enable visualization of even minimally fluorescent organs. ROIs were drawn by one of the investigators (LTR) around each organ, and a background region of the PLA plate, then those ROIs were applied to the original, unenhanced image (ImageJ, NIH; Supplementary Fig. 3). The average fluorescence intensity per pixel^2^ was quantified for each ROI on the original, unenhanced image (in arbitrary units per pixel^2^, or AU/p^2^). Average fluorescence of the background was subtracted from the mean fluorescence of each organ. The identical method was used to measure fluorescence from organs of mice given ^111^In-isotype-IR800 (*n* = 4 and 3 for each day, using the same mice as the gamma biodistribution experiment above).

To evaluate the practical use of the anti-GD2 tracer with widely available intraoperative tools, standard, non-IMI guided resection was conducted 4 days after tracer injection (*n* = 2). The intraoperative systems were then used to inspect for residual disease, and if identified, used to guide additional resection until no further disease could be detected.

### Tissue phantom studies

Tracer-bearing NB xenografts harvested from tumor-bearing mice for the biodistribution studies (*n* = 4) were implanted below 5 cm of processed tissue (Supplementary Fig. 5). Neoprobe^®^ and SPY-PHI handheld instruments were used to measure gamma decay and fluorescence from the surface of the overlying processed tissue with the removal of 5 mm increments of tissue, until the implanted xenograft was reached.

### Image processing and statistical analysis

Images for the fluorescence biodistribution were analyzed with ImageJ (NIH). Graphs were generated and statistical analyses were performed using Prism GraphPad v10. Organ uptake (%ID/g) in the biodistribution experiments is reported as mean ± SEM. Probe accumulation within organs was compared using one-way ANOVA, and if significant, the Sidak post-hoc test was used to compare the uptake of each organ to the tumor uptake. The tracer and isotype control experiments were run separately, so one-way ANOVA was used for each.

## Results

### Neuroblastoma cell lines express GD2

**Fig. 1:**
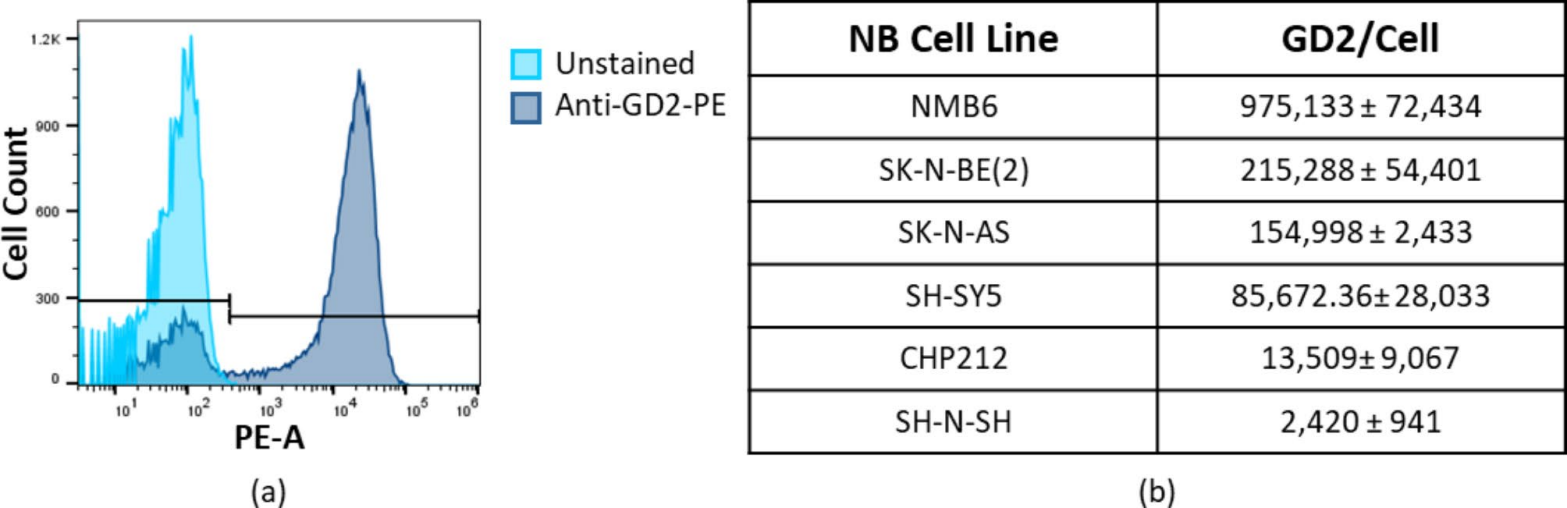
Human NB cell lines express GD2. (**a**) FACS histogram of unstained SK-N-BE(2) cells, unlabeled (left) versus labeled with PE-bound anti-GD2. (**b**) Quantification of average GD2 molecules per cell in human NB cell lines

Among six NB cell lines evaluated, the average GD2 molecules/cell varied widely (Fig. 1). As our prior imaging of myeloid cells in tumors demonstrated successful immunoPET with ~ 60,000 molecules/cell, we selected SK-N-BE(2) cells to establish xenograft models for subsequent studies, given its intermediate range among cell lines tested with an average of 215,288 ± 54,401 molecules of GD2 and its wide usage in preclinical research and established in vivo growth [30, 31]. 

### Tracer characterization

^111^In-aGD2-IR800 was synthesized with high radiochemical yield (> 90%) at the target molar activity (11.11 MBq/nmol). Substitution levels ranged from 0.75 to 2.0 DTPA chelators/antibody and 2–3 IRDye^®^ 800CW dyes/antibody, as determined by UV-Vis spectra comparing absorbance at 245 nm (DTPA) and 700 nm (IRDye^®^ 800CW) versus 280 nm (protein) (Supplementary Fig. 1). Optimization studies performed with more than 4 IRDye^®^ 800CW/antibody showed slight precipitation of the antibody. With fewer than 4 IRDye^®^ 800CW/antibody, no precipitation was observed on visual inspection and aggregation was not seen with SEC-HPLC (Supplementary Fig. 1). The binding affinities (K_d_) for anti-GD2 and DTPA-aGD2-IR800 were 2.39 nM (0.99–4.62 nM, 95% CI) and 21.31 nM (14.06–32.16 nM, 95% CI), respectively. These data support the feasibility of generating ^111^In-aGD2-IR800.

### Biodistribution analysis

Orthotopic SK-N-BE(2) xenografts were successfully grown in nude mice, reaching 907.5 ± 44.6 mm^3^ in volume (13.8 ± 0.24 mm in largest dimension) after 5 weeks of growth on MRI. In several mice, the left kidney was embedded within the tumor (Supplementary Fig. 2).

Specific accumulation of ^111^In-aGD2-IR800 or ^111^In-isotype-IR800 in GD2-expressing neuroblastoma tumors and non-target organs was examined by gamma decay count and fluorescence biodistribution analysis. All mice tolerated tracer administration well with no noted adverse effects. On both 4 and 6 days after probe injection, ^111^In-αGD2-IR800 showed greater tumor accumulation than almost all other measured organs, while corresponding isotype control tracer (^111^In-isotype-IR800) did not.

**Fig. 2:**
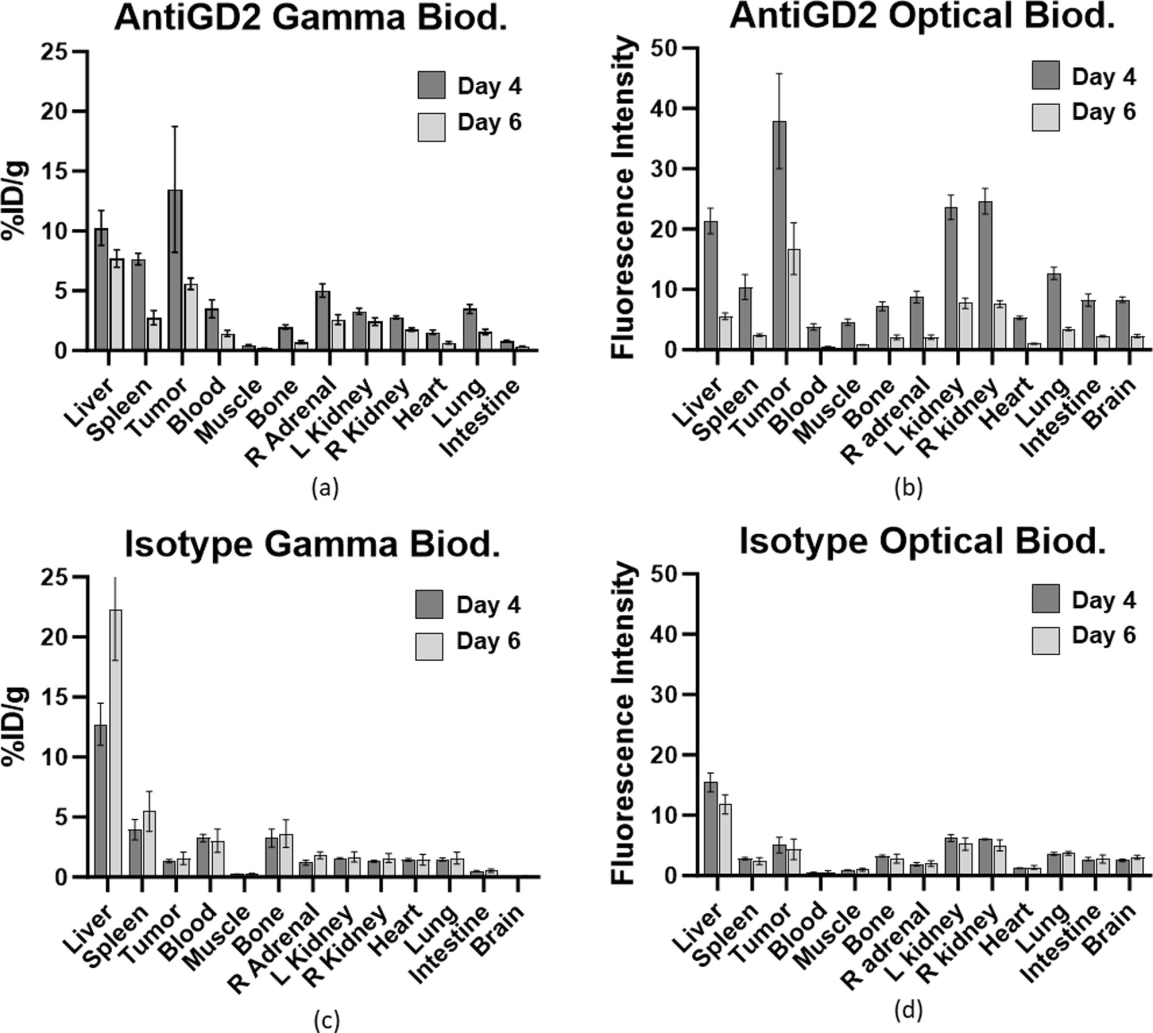
^111^In-αGD2-IR800 specifically accumulates in neuroblastoma. (**a**) Gamma decay of ^111^In-αGD2-IR800 is significantly higher in tumor than all other measured organs (*p* < 0.05) except for spleen and liver. (**b**) Fluorescence intensity of ^111^In-αGD2-IR800 is higher in tumor than all organs at both timepoints (*p* < 0.005). (**c**) Gamma and (**d**) optical biodistribution of ^111^In-isotype-IR800, shows tumor accumulation is significantly less than liver and spleen, with tumor-to-background ratios and differences dramatically lower than ^111^In-αGD2-IR800 at both timepoints

Gamma biodistribution analysis of ^111^In-aGD2-IR800 in NB-bearing mice 4 days after tracer injection (*n* = 7) demonstrated significantly higher uptake in the tumor (13.5 ± 5.27%ID/g) compared to blood (3.49 ± 0.75%ID/g; *p* < 0.001) and muscle (0.45 ± 0.06%ID/g; *p* < 0.0001) (Fig. 2a), with tumor-to-background (TBR) ratios of 3.87 and 30.0, respectively. As is common with IgG-based tracers, non-specific accumulation was noted in the liver (10.25 ± 1.45%ID/g; p = ns) and the spleen (7.66 ± 0.50%ID/g; p = ns), though accumulation was greater in the tumor than all other measured organs (*p* < 0.005). Similarly, six days after tracer injection (*n* = 6), tumor uptake (5.58 ± 0.48%ID/g) was significantly higher than all other organs (*p* < 0.0001) except the liver (7.70 ± 0.73%ID/g, *p* < 0.01), including the blood (1.44 ± 0.26%ID/g) and muscle (0.19 ± 0.01%ID/g) (Fig. 2a), with TBR ratios of 3.88 and 29.4, respectively.

Fluorescence biodistribution using the SPY-PHI camera and ImageJ analysis yielded similar results (Fig. 2b): On both days (*n* = 5 each), average fluorescence intensity was significantly higher in tumor (37.9 ± 7.88 AU/p^2^ on day 4 and 16.8 ± 4.30 AU/p^2^ on day 6) versus all other organs (*p* < 0.001, except day 4 vs. each kidney *p* < 0.01). On day 4, accumulation was low in the blood (3.80 ± 0.54 AU/p^2^) and muscle (4.57 ± 0.52 AU/p^2^), with tumor-to-blood and tumor-to-muscle differences of 34.1 and 33.3 respectively. Similarly on day 6, fluorescence was low in blood (0.43 ± 0.15 AU/p^2^), and muscle (0.8 +/- 0.045 AU/p^2^), with tumor-to-blood and tumor-to-muscle ratios of 16.37 and 16.0, respectively.

Isotype control (^111^In-isotype-IR800) accumulation in tumors, however, was low both by gamma and optical biodistribution analysis (*n* = 5 for each time point). Accumulation was similar in tumor (1.34 ± 0.87 on day 4; 1.56 ± 0.89%ID/g on day 6) and most other organs (p = ns) and was actually significantly lower than the spleen (day 4, *p* < 0.05) and the liver (both days, *p* < 0.0001) (Fig. 2c). Tumor-to-blood ratios of ^111^In-isotype-IR800 were 0.414 on day 4 and 0.514 on day 6 (9.3- and 7.5-fold lower than ratios in ^111^In-aGD2-IR800), while tumor-to-muscle ratios were 5.88 on day 4 and 6.32 on day 6 (5.1- and 4.7-fold lower than ^111^In-aGD2-IR800).

Similarly, tumor brightness was quite low after injection of ^111^In-isotype-IR800 (5.03 ± 1.3 AU/p^2^ on day 4 and 4.34 ± 1.69 AU/p^2^ on day 6; Fig. 2d). Four days after control tracer injection, fluorescence was still significantly higher in the tumor than the heart (1.24 ± 0.06 AU/p^2^; *p* < 0.001), blood (0.521 ± 0.06 AU/p^2^; *p* < 0.0001), muscle (0.89 ± 0.09 AU/p^2^; *p* < 0.001), and adrenal gland (1.88 ± 0.31 AU/p^2^; *p* < 0.01), but significantly lower than liver (15.4 ± 1.55 AU/p^2^; *p* < 0.0001). Six days after control tracer injection, tumor fluorescence remained was only significantly higher than blood (0.59 ± 0.20; *p* < 0.05) while much lower than liver (11.82 ± 1.59; *p* < 0.0001). The tumor-to-blood difference in fluorescence was also substantially lower with the isotype control tracer (4.51 and 4.14 on days 4 and 6, versus 34.1 and 16.37 with the ^111^In-αGD2-IR800). A similar decrease was in the tumor-to-muscle difference was seen (4.14 and 3.84 on days 4 and 6 versus 33.3 and 16.0 with ^111^In-αGD2-IR800).

### Handheld clinical IMI detection systems

**Fig. 3:**
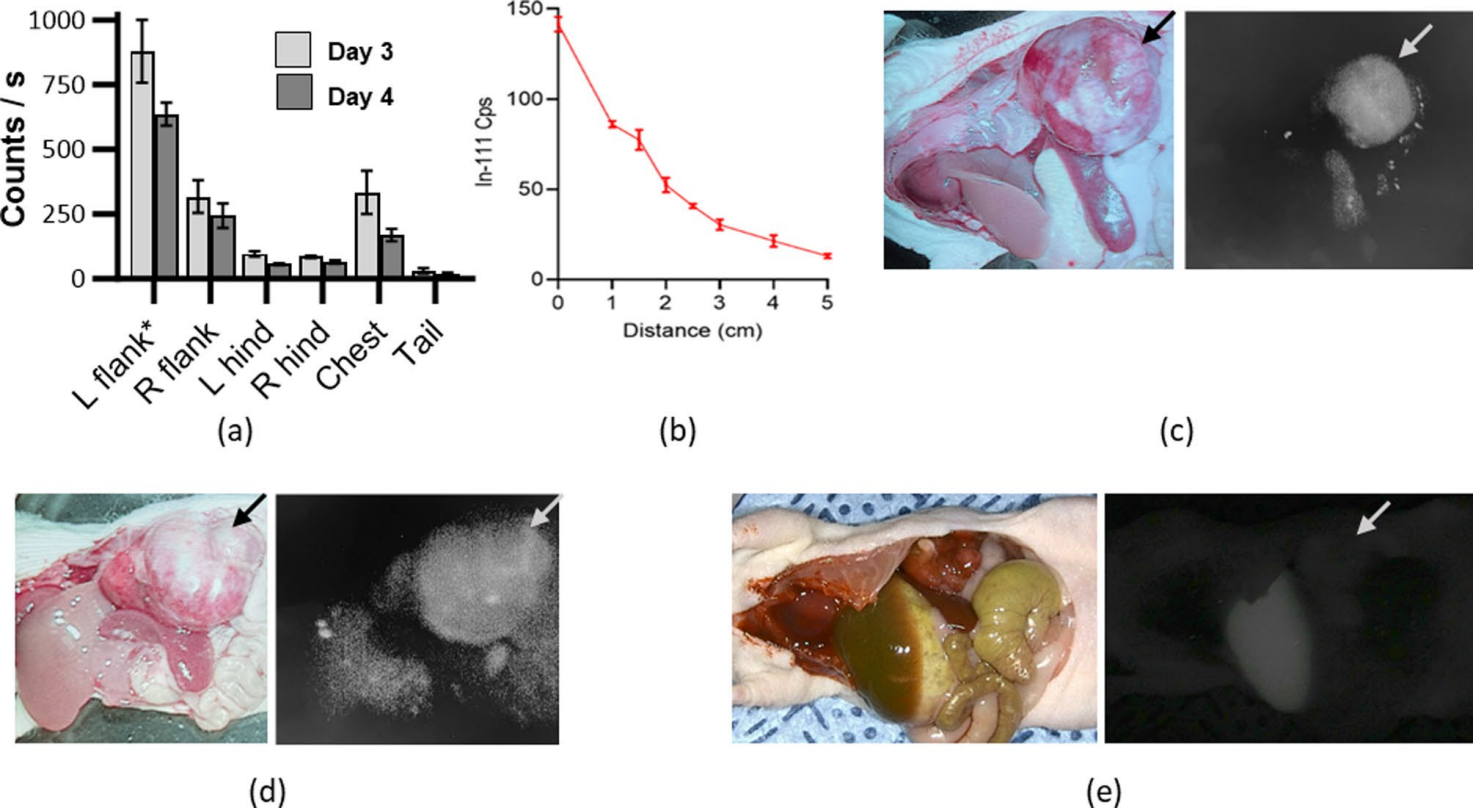
Handheld instruments can detect probe accumulation in the tumor. (**a**) Comparison of gamma decay of In-111 from different regions of the mouse body externally measured in situ with the handheld Neoprobe^®^, where the left flank contains the NB tumor and significantly greater decay (*p* < 0.0001). (**b**) Gamma counts from excised tumor embedded in increasing thicknesses of processed tissue (0.072 MBq). (**c**-**d**) Representative images of neuroblastoma xenografts taken with SPY-PHI camera in white light (left) and NIR (right), 4 days after ^111^In-αGD2-IR800 injection and (**e**) 4 days after ^111^In-isotype-IR800 injection

To evaluate tracer detection in vivo, NB-bearing mice received tail vein injections of ^111^In-αGD2-IR800 (4.8 MBq/50 µg in 100 µl DPBS; *n* = 4 using mice in the gamma biodistribution group). Three and four days later, gamma decay of body regions was measured percutaneously with the handheld Neoprobe^®^. At both time points, gamma decay signal was significantly higher in the tumor-bearing left flank than any other body region (Fig. 3a; *p* < 0.0001). Following laparotomy, fluorescence was subjectively evaluated with the handheld NIR camera, demonstrating strong qualitative fluorescence of the tumor with little surrounding signal (Fig. 3c and d; Supplementary Fig. 3b and c) while minimal tumor fluorescence was observable following similar isotype control tracer injection (Fig. 3e; Supplementary Fig. 3d, 3e, and 4).

Tumors harvested from mice at days 4 and 6 (*n* = 5 from gamma biodistribution group) were also implanted in processed tissue cubes to evaluate the effect of tumor depth on gamma and fluorescent signal detection. Gamma decay signal was detected from the surface of 5 cm of processed tissue and increased as 5 mm slices were removed from the tissue cube (Fig. 3b, Supplementary Fig. 5). Gamma signal was detected through 5 cm of tissue when the probe was aimed directly at the tumor, but no signal was detected when the probe was angled away more than ~ 5 degrees or moved laterally. Fluorescence could not be visualized until only 5 mm of processed tissue covered the tumor.

**Fig. 4:**
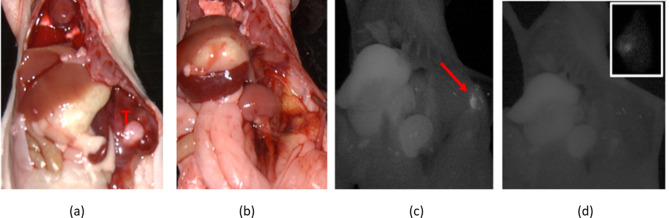
Fluorescence detection of residual tumor aids in complete resection. (**a**) Tumor is seen prior to attempted resection (white light; T denotes left adrenal tumor) while (**b**) no residual tumor was observed following resection. (**c**) Fluorescence imaging with the SPY-PHI camera demonstrated residual fluorescent tissue (arrow), which was then resected, (**d**) leaving no remaining fluorescent tissue in the tumor bed (inset: additionally resected fluorescent tissue)

The SPY-PHI camera also demonstrated efficacy in the detection of residual disease in and around the tumor bed after tumor resection. NB bearing mice (*n* = 2) were administered tracer 4 days prior to surgery, as prior biodistribution studies had demonstrated adequate TBR at that time. After laparotomy (Fig. 4a), complete tumor resection was attempted without IMI (Fig. 4b). Post-resection NIR imaging revealed residual fluorescence in the tumor bed, indicating likely residual disease (Fig. 4c) and was then used to remove the suspected residual disease (Fig. 4d; inset image of additionally resected tissue).

## Discussion

Multimodal therapy, including surgical resection, is crucial for the management of high-risk neuroblastoma (NB). Despite extensive therapy, 5-year survival remains only 40–50%, leading NB to be a disproportionately high cause of pediatric cancer mortality [2]. Incomplete macroscopic resection is associated with worse patient outcomes and maintenance immunotherapy is more effective with less residual disease [6, 32–34]. Achieving an adequate degree of resection safely, however, is challenging, as these tumors are not well circumscribed, develop an intimate relationship with vital structures that must be preserved, and can form satellite lesions that are difficult to find. With only direct tumor visualization and palpation for guidance, up to 30% of resections for high-risk NB are inadequate, and up to 50% lead to complications [6, 32, 34, 35]. IMI is an emerging strategy to specifically identify and visualize tumors, to facilitate a safer, more complete resection and thus, potentially improve post-operative outcomes, though it has not previously been used for pediatric cancer indications. In this study, we demonstrate the utility of our dual-labeled GD2-specific tracer to localize and visualize NB with commonly used intraoperative tools.

GD2 was selected as the IMI target because it is overexpressed in NB, as well as several other cancers, and has minimal expression on normal tissues. In fact, due to these features, the NIH has identified GD2 as one of the most critical tumor antigens for targeted therapies [5, 20–22, 36]. While radio-guided surgery (RGS) and fluorescence-guided surgery (FGS) have proven benefit, each technique has shortcomings overcome by the other [20, 37–39]. Consequently, we have chosen to dual-label our IMI agent, to enable detection of deeper or occult tumors (RGS) and tumor margin visualization (FGS). Therefore, DTPA-αGD2-IR800 was synthesized and radiolabeled with Indium-111 to evaluate it as an IMI agent in a mouse model of NB. While the binding affinity of DTPA-aGD2-IR800 is just under 10-fold lower than unlabeled antibody (21.31 nM versus 2.39 nM), it remains adequate for intraoperative imaging, based on other targeted antibody-based tracers [40]. The number of IRDye^®^ 800CW and DTPA per molecule will also be adjusted in future work to optimize binding and signal. While tracer was tolerated well by all mice, pharmacology and toxicity studies must be conducted in the future.

Both gamma and fluorescence biodistributions demonstrated excellent antigen-specific accumulation of the tracer in tumor tissue, with some non-specific accumulation in the liver and spleen, as seen with other antibody-based IMI tracers. After 5 weeks of growth, the tumors used in these studies were an average of907.5 ± 44.6 mm^3^ in volume (13.8 ± 0.24 mm in largest dimension). In gamma biodistribution studies, the tracer had excellent tumor accumulation (13.5 ± 5.27%ID/g on day 4 and 5.58 ± 0.48%ID/g on day 6), tumor-to-blood ratios (3.9), and tumor-to-muscle ratios (30) (Fig. 2a). Similar results were seen in the fluorescence biodistribution, where the average fluorescence intensity of tumor (37.9 ± 7.88 AU/p^2^ and 16.8 ± 4.30 AU/p^2^ on days 4 and 6) was significantly higher than all other measured organs on both days (*p* < 0.001; Fig. 2b). Tumor-to-blood differences (31.1 AU/p^2^ on day 4 and 16.37 AU/p^2^ on day 6) and tumor-to-muscle differences (33.3 AU/p^2^ on day 4 and 16.0 AU/p^2^ on day 6) enabled excellent NIR fluorescence visualization of tumors. Fluorescence biodistribution was performed using the handheld SPY-PHI instrument, which is currently used clinically, rendering the results particularly relevant for surgical use of the tracer. Furthermore, the tumor was detectable in vivo with the Neoprobe^®^ and SPY-PHI NIR camera (Fig. 3), both widely available handheld intraoperative instruments.

The isotype control tracer had very low tumor accumulation on both days (1.34 ± 0.15%ID/g and 1.56 ± 0.52%ID/g) and was not significantly higher than any other organ (Fig. 2c). While fluorescence was higher in the tumor versus some background organs, it remained overall quite low (5.03 ± 1.3 AU/p^2^ on day 4 and 4.34 ± 1.69 AU/p^2^ on day 6; Fig. 2d) and was significantly lower than the liver (Supplementary Fig. 3d, 3e, and 4). This indicates antigen-specific binding and accumulation of the anti-GD2 probe in the tumor, rather than accumulation due to the enhanced permeability and retention (EPR) effect.

To study IMI performance, the Neoprobe^®^ and SPY-PHI tools were used in situ after tracer injection. Gamma decay detection with the handheld Neoprobe^®^ was higher in the tumor-bearing left flank than in other body regions (Fig. 3a; *p* < 0.0001) and strong qualitative fluorescence of the tumor was seen (Fig. 3c and d; Supplementary Fig. 3b and c). Furthermore, the effect of tumor depth on detection was assessed with a processed tissue phantom model. Using conventional surgical methods, identifying small tumors below a tissue or organ surface can prove extremely challenging. Gamma detection of the labeled tumor was excellent through up to 5 cm of intervening processed tissue (Fig. 3b). Gamma detection provided directional guidance, required by surgeons to continue dissection, as counts were highest when the probe was on an axis pointed directly towards the tumor. By comparison, fluorescence was significantly limited by depth, only visible through 5 mm or less of tissue, consistent with previously reported depth limitations of FGS [14, 15]. 

To further assess ^111^In-αGD2-IR800-based IMI, mice with xenograft NB tumors were injected with the tracer and complete tumor resection was faithfully attempted without IMI guidance (standard surgery). Imaging with the SPY-PHI, however, revealed presumed residual disease that had not been appreciated during the initial white-light resection. The residual fluorescent tissue was then resected under IMI guidance (Fig. 4). Future work will include pathologic evaluation of the additional tissue resected by IMI guidance to further assess the specificity of the targeted tracer in vivo. We will also utilize our newly established rodent renal-sparing radical adrenalectomy survival surgery model, to compare safety, degree of resection, tumor recurrence, and survival following resection, with or without use of ^111^In-αGD2-IR800-based IMI [41]. 

Overall, use of this probe in GD2-expressing cancer demonstrated an excellent tumor-to-background signal using intraoperative gamma and NIR hand-held detectors, enabling visualization and removal of suspected residual tumor tissue in mice with NB orthotopic xenograft tumors. Other IMI tracers are limited in their use for resection of neuroblastoma. Wellens et al. previously generated an anti-GD2-IRDye800CW IMI tracer, which is fluorescent only and thus has a more limited depth of detection than the dual-labeled tracer [42]. Both our dual-labeled tracer and the Wellens fluorescent tracer have non-specific liver accumulation, which could limit the efficacy in detecting and visualizing liver metastases.

There are three currently FDA-approved fluorescent tracers for cancer (5-ALA, Cytalux or OTL38, and Luminex or LUM015), which similarly have limited depth of penetration. No dual-labeled tracers, which enable both fluorescence visualization, and radioactivity-based depth of detection are FDA-approved and those in clinical trials target carbonic anhydrase IX, gastrin-releasing peptide receptor, and CEA, which are not as uniformly and highly expressed in neuroblastoma [17, 19, 43, 44]. Collectively, our data demonstrate the potential to improve the safety and completeness of NB detection and resection using IMI with ^111^In-αGD2-IR800.

While the murine anti-GD2 antibody used in this study could be immunogenic in humans, it is an isotype variant of the FDA-approved chimeric antibody, Dinutuximab, which shares the same variable regions (V_H_ and V_L_) and thus establishes the utility of the anti-GD2 targeting strategy. We anticipate generating a similar agent with Dinutuximab which, even at higher doses and in combination with immunomodulatory drugs IL-2 and GM-CSF, is rarely immunogenic in humans [45]. The tracer will be further optimized for binding and fluorescence by adjusting the number of DTPA and IRDye^®^ 800CW per antibody. Prior studies are encouraging that a single dose of Dinutuximab-based tracer for IMI, which is given without immunomodulatory agents and correlates with a human dose approximately 40% of the daily therapeutic dose, should have little effect on future therapy, though this will be important to confirm [46]. Because the mouse body is small relative to the size of the Neoprobe^®^, there may have been overlapping signal from the tumor, liver, and spleen *in situ.* Our orthotopic xenograft rat model should improve spatial separation of tumor and abdominal organs [46]. 

While there have been recent advances in tumor-specific antigen tracers in adult oncology, their utility in pediatric oncology remains limited [47, 48]. We have developed a functional IMI tracer that is detectable with excellent sensitivity and specificity by widely available surgical instruments. This novel IMI-based approach may facilitate safer and more complete resection of NB in children, with a potential survival benefit.

## Conclusions

We present a novel intraoperative imaging agent to enable safer and more complete operative resection by enhancing the targeted detection of tumor using tools already in regular clinical use. Our dual-labeled anti-GD2 tracer, ^111^In-αGD2-IR800, demonstrated antigen specificity and allowed specific detection and visualization of neuroblastoma in an animal model. This IMI tracer could provide a means to not only increase the completeness of resection, but also to minimize and potentially prevent surgical complications, thereby improving patient outcomes.

## Electronic supplementary material

Below is the link to the electronic supplementary material.

Supplementary Material 1

## Data Availability

The datasets used and/or analyzed during the current study are available from the corresponding author on reasonable request.

## Abbreviations

FGS: Fluorescent-guided surgery
IMI: Intraoperative molecular imaging
NB: Neuroblastoma
NIR: Near infrared
RGS: Radio-guided surgery
ROI: Regions of interest
